# Quantitative Image Analysis of Axonal Morphology in In Vivo Model

**DOI:** 10.3390/mps6060116

**Published:** 2023-12-01

**Authors:** Laurie Nemoz-Billet, Jacques Brocard, Florence Ruggiero, Sandrine Bretaud

**Affiliations:** 1 Institut de Génomique Fonctionnelle de Lyon, Ecole Normale Supérieure de Lyon, UMR5242 CNRS, Université Claude Bernard-Lyon-1, 69364 Lyon, France; laurie.nemoz-billet@ens-lyon.fr (L.N.-B.); florence.ruggiero@ens-lyon.fr (F.R.); 2 PLATIM, SFR Biosciences, ENS de Lyon, Inserm US8, CNRS UMS3444, Université Claude Bernard-Lyon-1, 69364 Lyon, France; jacques.brocard@ens-lyon.fr

**Keywords:** quantification, 2D analysis, axon length, branching, motor neuron, development, zebrafish

## Abstract

Quantifying axonal branching is crucial for understanding neural circuit function, developmental and regeneration processes and disease mechanisms. Factors that regulate patterns of axonal arborization and tune neuronal circuits are investigated for their implication in various disorders in brain connectivity. The lack of a reliable and user-friendly method makes the quantitative analysis of axon morphology difficult. Specifically, methods to visualize and quantify the complex axon arborization are challenging to implement and apply practically. Our study was aimed at developing a robust but simple method of quantification that used ImageJ 2D analysis and compared it with Imaris visualization and analysis of 3D images. We used zebrafish fluorescent transgenic lines to perform in vivo imaging of developing motor neuron axons that adequately reflected the complexity of axonal networks. Our new method, developed on ImageJ, is easy and fast, giving access to new information such as collateral distribution along the axonal shaft. This study describes step-by-step procedures that can be easily applied to a variety of organisms and in vitro systems. Our study provides a basis for further exploration of neural circuits to gain new insights into neuronal disorders and potential therapeutic interventions.

## 1. Introduction

Neurons display various morphologies as they develop complex axonal collaterals and terminal arbors in order to establish synaptic connection and communicate. As such, axonal branching pattern participates in neuronal function. In chickens, motor neurons that innervate slow and fast muscles display different branching morphologies, leading to different physiological properties [1]. The importance of axonal branching was also emphasized in studies showing that the morphology of neurons used to model neurological disorders, such as autism or spinal muscular atrophy, is altered in different organisms [2,3]. Axons form complex networks that make the quantification of potential defects in axonogenesis, as well as in nerve regeneration, difficult, specifically in model organisms. In general, analysis and quantification of neuron morphology and axon branching are adequately performed using the Imaris licensed software either in zebrafish [4,5] or in other model systems, notably drosophila and cultured cells [6]. However, there are important limitations in using it. First, a license considerably limits who can use the program, and second, the methods to process the images and quantify morphology and branching are not described in sufficient detail to make it an easy-to-follow protocol. The latter statement also applies to the use of the free ImageJ software [2,7,8]. There is thus a need to develop a free and user-friendly quantitative method and to provide the scientific community with a step-by-step protocol for rapid and easy analysis and quantification of axonogenesis in in vivo and in vitro models.

In the present study, we used zebrafish as an animal model to analyze axonal morphology and branching of motor neurons during embryonic development. This small vertebrate organism has been extensively used to study axonogenesis over the last two decades [9]. Zebrafish embryos are perfectly amenable for live imaging due to their small size and their optical transparency. The embryonic development is fast, and motor neurons can be easily visualized in vivo thanks to the creation of transgenic fluorescent reporter lines [10]. As such, several studies have reported alterations of axon morphogenesis associated with gene knockdown or knockout in developing zebrafish [4,5,7]. This increases the need to develop methods to accurately quantify these defects, specifically in case of minor defects. This is not an easy task, as zebrafish already display a quite complex axonal arborization after only a few days of development.

Using motor neuron images of live zebrafish embryos, we developed a procedure to automatically extract data from 2D images in order to analyze and quantify axon morphology using ImageJ free software. We also described how to use the software dedicated to 3D neuron analysis (Imaris, bitplane), and we compared both methods. First, the quantitative image analysis method developed in this study is easy to use and requires only a few minutes per image to obtain measurements of key parameters, such as the axon length and the branching density. Second, it allows the quantification of these parameters in an unbiased manner, as there is no need to trace collaterals manually, as is the case with the Imaris software and other ImageJ plugins. Third, our ImageJ script provides additional important information, such as the collateral distribution along the axonal shaft. Finally, the new method described in this study can be applied to in vitro models and different types of neurons, and by extension, to branching morphogenesis.

## 2. Materials and Methods

### 2.1. Zebrafish Line and Breeding

Zebrafish maintenance and embryo collection were performed at the zebrafish PRECI facility (Plateau de Recherche Expérimentale de Criblage In vivo, UMS CNRS 3444 Lyon Biosciences, Gerland, France) in compliance with French government guidelines. Embryos obtained from natural spawning were raised following standard conditions. The developmental stages were given in the hours post-fertilization (hpf) at 28.5 °C according to morphological criteria [11]. Tg(mnx1:gfp)ml2 transgenic embryos were used to directly visualize motor neurons. AB-TU embryos at one cell-stage were used for plasmid injection. Phenylthiourea (PTU, 0.21 mM, Sigma-Aldrich, St. Quentin Fallavier, France, P7629) was added at 24 hpf to prevent pigmentation.

### 2.2. Embryo Manipulation and Immnostaining

AB-TU embryos were injected at the 1-cell stage with 50 pg of *Shh* mRNA, as described in Guillon et al., 2016 [12]. Immunostaining with znp-1 antibody (Hybridoma bank, Iowa City, IA, USA) was performed at 26 hpf (1/20, Hybridoma bank) to reveal primary motor axons, as previously described (Guillon et al., 2016) [12]

### 2.3. Confocal Acquisition and Image Post-Processing

Five live embryos of 24 and 48 hpf were used. They were anesthetized with 0.016% tricaine (Ethyl 3-aminobenzoate, Sigma-Aldrich, St. Quentin Fallavier, France E10505) before being laterally mounted into 4-well Ibidi dishes (Biovalley/CliniSciences group, Nanterre, France 80426) with 1% low melting agarose (ThermoFisher Scientific/Life Technology SAS, Illkirch-Graffenstaden, France, 16520050). E3 medium (5 mM NaCl, 0.17 mM KCl, 0.27 mM CaCl2, 0.33 mM MgSO4) containing 0.016% tricaine was added above the low melting agarose layer to maintain a humid environment. Embryos were observed and imaged over a 4 somite-region at the level of the yolk sac extension using an inverted confocal microscope (Zeiss LSM 780, Oberkochen, Germany). For each embryo somite, Zen software v.3.6 post-processing (image subset, Zeiss) was used to manually select a ROI corresponding only to the motor axon and not cell bodies.

### 2.4. ImageJ Analysis

**Figure 1:**
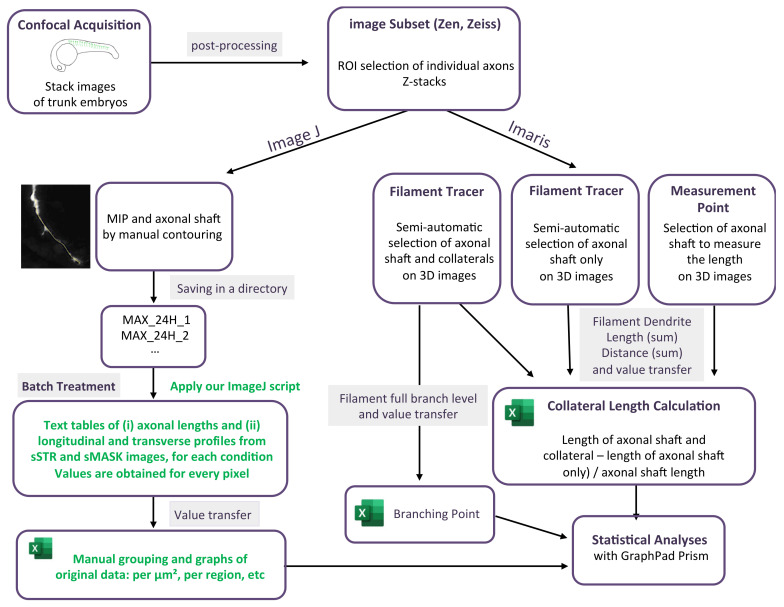
Workflow chart of the two methodologies used to quantify axonal length and arborization. The green font corresponds to application of our script.

For each stack of images corresponding to a single motor neuron axon, axonal contouring was laid out manually on a maximum intensity projection image (MIP). A series of instructions gathered in an ImageJ macro (https://github.com/jbrocardplatim/Axon-Branching-Zebra, accessed on 30 October 2023) allowed for accessing the axonal length and a straightened view of the axonal shaft and its collaterals, within a 25 µm width (STR). Each image was then thresholded and skeletonized in order to produce only mask images (MASK) of collateral extensions that were longer than 0.5 µm. STR or MASK images from the same time point (24 hpf or 48 hpf) were stacked together to produce sSTR or sMASK stacks; average and maximum intensity projections for each were shown. Finally, a 5-µm-wide longitudinal band, corresponding to the axonal shaft, was removed from each image before calculating an average intensity for each, either transversally or longitudinally. The detail of the script can be found following this link: https://github.com/jbrocardplatim/Axon-Branching-Zebra/blob/main/MacroAxonesZebra_v10%20-%20review.ijm, accessed on 30 October 2023. A detailed workflow chart of the image analysis is presented in Figure 1.

### 2.5. Raw Data Processing

Tab-separated arrays of mean values obtained for transverse and longitudinal profiles as described above were also produced by the ImageJ macro described elsewhere (https://github.com/jbrocardplatim/Axon-Branching-Zebra, accessed on 30 October 2023). However, the pixel-to-pixel variability being huge (see individual profiles in Figures S1 and S2), it was decided to average collateral numbers within a meaningful unit = µm^2^. Hence, collateral numbers/µm^2^ were determined as the sum of collaterals for 4 pixels in the x direction (representing 0.9932 µm) and normalized by pixel size in the y direction. More specifically, mean fluorescence intensity/µm^2^ were calculated from intensity (=STR) images whereas collateral numbers/µm^2^ were calculated from binary (=MASK) images. For these images, 0–1 binary masks were averaged along (i) the *x*-axis = longitudinal profile per µm in the y-direction and (ii) the *y*-axis = transverse profile per µm in the x-direction.

### 2.6. Imaris Analysis

A filament tracer module from Imaris software (v7.7, Bitplane, Zurich, Switzerland) was used to measure axon branching (collaterals and terminal arborization) in individual somites. Semi-automatic detection with the autopath algorithm was used to track the axon. In statistics options, filament dendrite length (sum) was selected to obtain the total length in µm of the selected element. The length of the axonal shaft (until terminal arborization) was then manually selected from the filament tracer axonal shaft image (in 3D) by using the measurement point module. The length of the axonal shaft was subtracted from the total axonal tree to determine the length of the only axonal branching. The data were then normalized with the length of the axon measured with the measurement points in Imaris. The number of branching points was selected and measured using the statistic in Imaris. The detail of the analysis is graphically presented in Figure 1.

### 2.7. Statistical Analysis

Statistical analyses were carried out using GraphPad Prism (v6) on a total of 19 motor axons at 24 hpf and 20 at 48 hpf, from 5 different embryos for each developmental stage. In experiments using embryos that were *Shh*-injected or embryos with an empty plasmid, a total of 12 motor axons were analyzed from 3 different embryos for each condition. Normality was tested using the Shapiro–Wilk test followed by the Student *t*-test for normal distribution. For non-parametric data, multiple Mann–Whitney tests were applied and false discovery rate-corrected (q = 5%) using GraphPad; medians with minimum and maximum values were represented.

## 3. Results

### 3.1. Model of the Study

**Figure 2:**
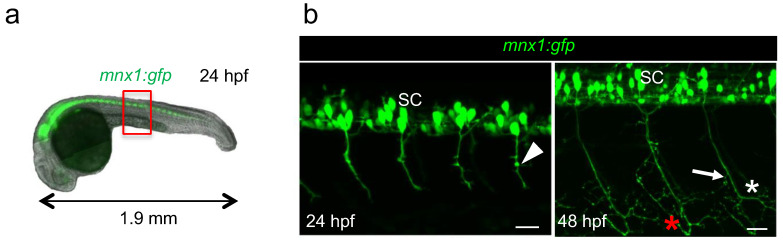
Visualization of motor neurons in developing *mnx1:gfp* zebrafish embryos at 24 hpf and 48 hpf. (**a**). Fluorescence image of a 24 hpf transgenic embryo showing the motor neurons in the spinal cord (in green). Red box indicates the exact region where motor axons were imaged, at the level of yolk sac extension. (**b**) Four-somites images of the trunk of a 24 hpf (**left**) and 48 hpf (**right**) *mnx1:gfp* embryo used for quantification; motor neurons and their CaP axons are in green. SC, spinal cord. Arrowhead, minor collateral; arrow, larger collateral; red and white asterisks point to axonal growth along the vertical myoseptum and arborization, respectively. Anterior is left, lateral views are shown. Scale bars = 25 µm.

The sequence of motor neuron development has been originally studied in zebrafish using dextran red injection to trace axons [13]. Here, we used live *mnx1:gfp* transgenic embryos in which we can directly visualize motor neuron axons all throughout the development with fluorescence microscopy (Figure 2a). We decided to image axons of caudal primary motor neurons, referred to as CaP, because they are the first to exit the spinal cord and they are easily visualized as they grow ventrally. CaP axons are also the first to branch [14]. The two developmental stages chosen for this study were 24 hpf (hours-post-fertilization) and 48 hpf, when axon arborization complexified (Figure 2a,b) [10]. At 48 hpf, additional axons of the secondary motor neurons that followed the same path pioneered by primary motor neurons were observed, as previously described [9] (Figure 2b). Indeed, while only minor collaterals were observed at 24 hpf (Figure 2b, arrowhead), at 48 hpf, axon terminals exhibited extensive arborization (Figure 2b, asterisks) and most of the collaterals were located in the distal segment of axons (Figure 2b, arrow). The bifurcation observed at the level of the ventral myotome indicated that the axon grows along the vertical myoseptum to innervate lateral muscle fibers (Figure 2b, red asterisk). For quantification, we generated motor axon images from four individual somites at the level of yolk extension (Figure 1a, red box) using Zen software to minimize the intrinsic variability due to embryo development. The resulting stacks of images correspond to motor neurons axons without cell bodies (see Materials and Methods for details).

### 3.2. Branching Quantification Using a Home-Made Script with ImageJ

**Figure 3:**
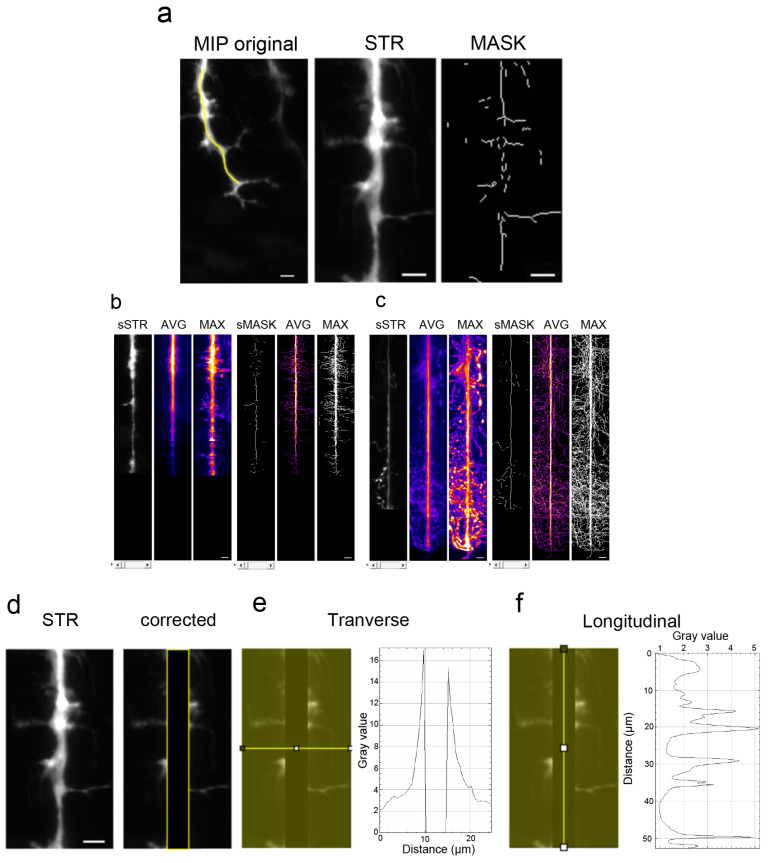
Method for axonal branching quantification with a new ImageJ script. (**a**) Axonal contouring was performed manually on a maximum intensity projection image (MIP) of each motor axon, then straightened to form a new image of the axonal shaft and its collaterals (STR) and a mask image after automatic thresholding and skeletonization (MASK). (**b**) Stacks of straightened (sSTR) and mask images (sMASK) obtained with 24 hpf axons, as described in (**a**), as well as averaged (AVG) and maximal (MAX) intensity projections of said stacks. (**c**) Stacks of straightened (sSTR) and mask images (sMASK) obtained from 48 hpf axons, as described in (**a**), as well as averaged (AVG) and maximal (MAX) intensity projections of said stacks. Only one image from the stack is presented as an example (sSTR and sMASK) (**d**). Removal of the central axonal shaft before automatic transverse (**e**) or longitudinal (**f**) mean profiling. Scale bars = 5 µm.

We developed a new script using Image J that is free to download from GitHub in order to quantify the distribution of collaterals along the axonal shaft. After manual selection of the axonal shaft from all maximum intensity projection 2D images (Figure 3a, MIP original), a straightened view of the axonal shaft and its collaterals within 25 microns was generated (Figure 3a, STR). In order to further quantify collaterals, automatic detection and thresholding allowed for the production of binary mask images of collaterals longer than 0.5 µm (Figure 3a, MASK). For both stages (Figure 3b, 24 hpf and Figure 3c, 48 hpf), STR and MASK images were aligned to obtain stacks, named sSTR and sMASK, respectively. The average intensity (Figure 3b,c, AVG) and maximum intensity projections (Figure 3b,c, MAX) of the stacks were then displayed. From these representations, we observed that arborization and collaterals extended drastically from 24 hpf to 48 hpf (see Figure 3b,c, MAX projections). Moreover, ventral arborization appeared more developed than dorsal arborization at 48 hpf (Figure 3c, compare lower to upper parts of AVG or MAX projections).

To quantify these observations, we extracted a transverse (Figure 3e) and a longitudinal (Figure 3f) mean profile from each image after a 5 µm wide longitudinal band was removed to discard the main axonal shaft (Figure 3d, corrected). Both types of profiles, generated from STR images of fluorescence intensity images, were plotted individually or together for statistical comparisons (Figure S1). Although significant differences were calculated between the intensity profiles at 24 hpf and 48 hpf (Tables S1–S4), one may wonder whether a global surge of intensity may interfere with such measurements. Hence, we decided to use transverse and longitudinal mean profiling of binary MASK images to further quantify collateral development.

**Figure 4:**
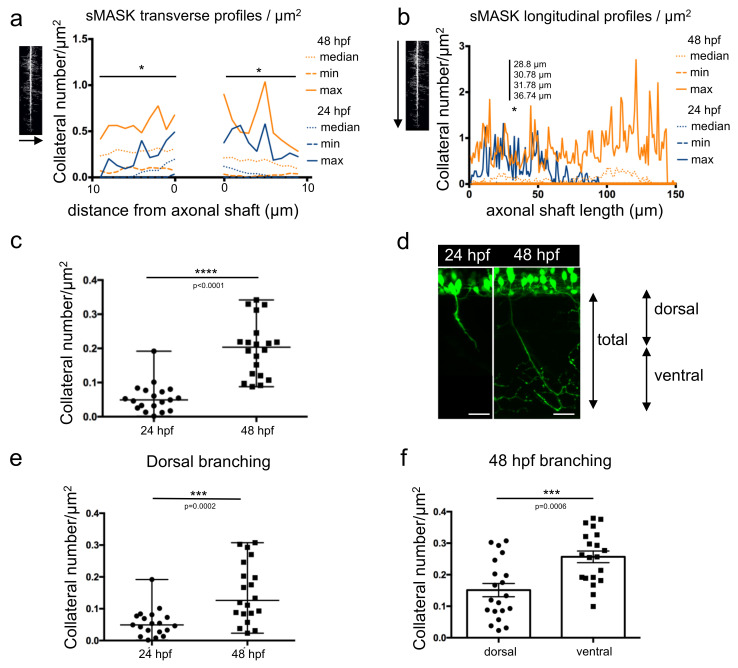
Measurement of axonal branching in 24 hpf and 48 hpf zebrafish embryos. (**a**) Median, min and max values obtained for number of collaterals/µm^2^ from the transverse profile of 19 mask images of 24 hpf axons (dark blue) and 20 mask images of 48 hpf axons (orange). *, significantly different profiles using Mann–Whitney tests corrected for multiple comparisons (individual *p* values in Table S7). (**b**) Median, min and max values obtained for number of collaterals/µm^2^ from the longitudinal profile of 19 mask images of 24 hpf axons (dark blue) and 20 mask images of 48 hpf axons (orange). *, significantly different profiles using Mann–Whitney tests corrected for multiple comparisons (individual *p* values in Table S8). (**c**) Total branching quantified in both stages along the full length of the axon. Statistical analysis was performed using Mann–Whitney test. Branching in normalized to axon length. **** *p* < 0.0001. (**d**) Annotation of dorsal and ventral regions to the corresponding developmental stages. (**e**) Branching quantified in the dorsal, proximal part of the axon over a length of 70 µm at both stages. Statistical analysis was performed using Mann–Whitney test. Branching is normalized to axon length.*** *p* < 0.001. (**f**) Branching in two different regions of the axon (first 70 µm, dorsal part and the remaining ventral part) compared in 48 hpf embryos. Statistical analysis was performed using Student *t*-test. Branching is normalized to axon length. *** *p* < 0.001. Error bars are SEM.

Both types of profiles were plotted individually (Figure S2) or together for statistical comparisons (Figure 4a,b and Tables S5–S8). Transverse profiles displayed statistically significant differences between the development of collaterals at 24 hpf and 48 hpf, at almost every position (Figure 4a and Table S7). In contrast, in the longitudinal profiles, only four points located between 28 and 36 µm away from the top showed a significantly higher number of collaterals at 48 hpf vs. 24 hpf (Figure 4b and Table S8).

Total branching along the whole axon length was determined in 24 hpf and 48 hpf embryos (Figure 4c). A statistically significant higher amount of branching was observed at 48 hpf compared to 24 hpf, as expected. Axonal shafts were then arbitrarily divided into two regions: dorsal and ventral (Figure 4d). The dorsal region encompassed the proximal part of the axon over a 70 µm length and corresponded to an average of axonal length reached after 24 h of development. The ventral part corresponded to the remaining proximal region along the axonal shaft. When comparing the axon dorsal parts in 24 hpf and 48 hpf embryos, quantification revealed a significant increase in collateral numbers at the later developmental stage (Figure 4e). Within the same 48 hpf embryos, the axon ventral parts displayed a statistically significant higher number of collaterals (Figure 4f) in agreement with the results showed in Figure 2b.

**Figure 5:**
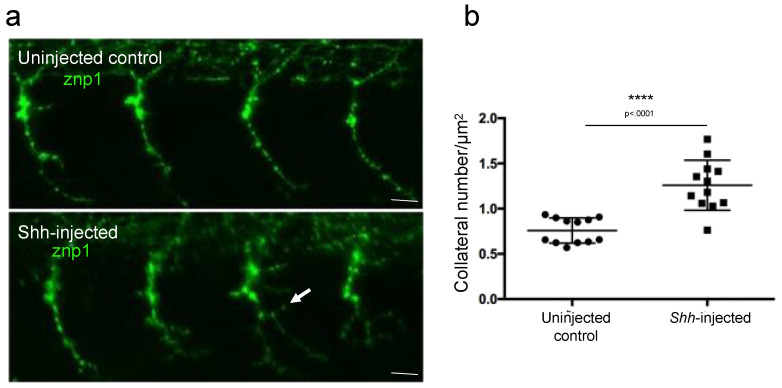
Measurement of axonal branching in 26 hpf zebrafish embryos injected or not injected with *Shh* mRNA. (**a**) Motor neuron axons are immunostained with znp-1 antibody. Arrow, aberrant branching. (**b**) Quantification of the total branching along the full length of the axons. Statistical analysis was performed using a Mann–Whitney test. Branching is normalized to axon length. **** *p* < 0.0001. Error bars are SEM. Scale bars = 15 µm.

In order to test whether our new developed ImageJ script was able to discriminate more subtle axonal branching defect phenotypes, we measured the axon arborization in 26 hpf control embryos or embryos injected with Shh mRNA after labeling the motor axon with znp-1 antibody (Figure 5). Shh is a powerful morphogen known to trigger the differentiation of slow muscle fibers in zebrafish [15]. Overexpression of Shh in developing embryos provoked the conversion of the entire myotome into slow muscle fibers [12,15]. We thus reasoned that overexpression of Shh should result in defects in motor neuron axon growth, a defect that has not been described yet. Aberrant branching was indeed observed in *Shh*-injected embryos using our method of image analysis and quantification (Figure 5a). The quantification of the branching phenotype demonstrated a statistically significant increase in the total branching in Shh-injected embryos compared to uninjected control embryos (Figure 5b).

### 3.3. Branching Quantification Using 3D Analysis Imaris Software

**Figure 6:**
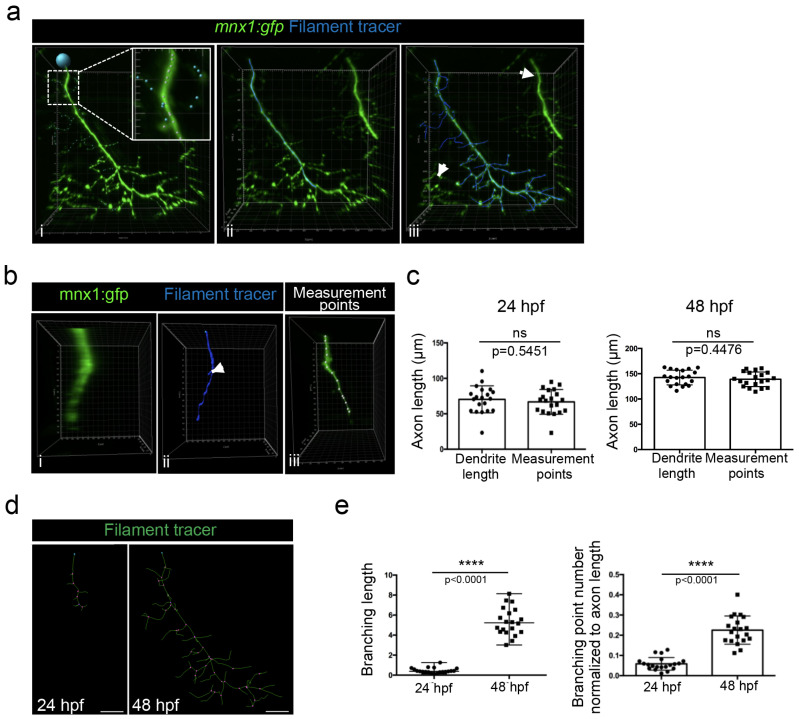
Method for axonal branching quantification in 24 and 48 hpf mnx1:gfp zebrafish embryos using Imaris software. (**a**) Semi-automatic selection of motor neuron axon branching in individualized nerve of a 48 hpf embryo, using filament tracer from Imaris. (i) Starting point is represented by the blue point. Higher magnification in white square shows seed points. White seed points correspond to automatically detected branching and blue seed points to the manually added one. Axonal shaft is selected in blue. (ii) Axonal shaft and all collaterals are selected in blue. (iii) Collaterals of adjacent nerves indicated by the arrow are excluded from the selection. (**b**) Visualization of a motor axon from a 24 hpf embryo after rotation of 90 degrees to show its thickness (i) and its 3D reconstruction with filament tracer (ii). The same axon was analyzed with measurement points module (iii). (**c**) Quantification of axon length with dendrite length and measurement points methodologies in 24 hpf (n = 19) and 48 hpf (n = 20) embryos. Statistical analysis was performed using a Student *t*-test. ns: non-significant. Error bars are SEM. (**d**) 3D reconstruction of motor axons in 24 hpf and 48 hpf embryos using filament tracer module of Imaris. The blue point corresponds to the starting point of the axon. The pink points correspond to branching points. Lateral views. (**e**) Quantification of the total branching length normalized to axon length in 24 hpf (n = 19) and 48 hpf (n = 20) embryos. Statistical analysis was performed using a Mann–Whitney non-parametric test. **** *p* < 0.0001. Quantification of the number of branching points normalized to axon length in 24 hpf (n = 19) and 48 hpf (n = 20) embryos. Statistical analysis was performed using a Student *t*-test. Error bars are SEM. Scale bars = 20 µm.

Because we noticed that a detailed method for Imaris software was surprisingly missing in the literature, we decided to describe here a step-by-step method to track axonal shaft and collaterals in order to (1) perform a 3D reconstruction and (2) quantify axonal branching. The filament tracer analysis module of Imaris software allowed the 3D reconstruction and the analysis of neurons, as well as the arborization of dendrites and spines. We used the semi-automatic detection of the autopath algorithm of the filament tracer module to trace axons. The starting point of the axon at the top of the image was manually selected (Figure 6a(i), blue circle). The seed point threshold was adjusted in order to detect either only the axonal shaft (Figure 6a(ii)) or the axonal shaft with all branchings (Figure 6a(iii)). Statistical dendrite length (sum) was used to determine each length (axonal shaft and all branchings); the length of the axonal shaft was subtracted from the total axonal tree to determine the length of the axonal branching only and was normalized to the axon length.

The seed points that detected branches of motor nerves of the neighboring myotomes were manually removed (Figure 6a(ii), arrows). Conversely, extra seed points were manually added in order to detect collaterals of lower fluorescent intensity (Figure 6a(i), blue seeds in higher magnification).

Due to the axon thickness, especially in 24 hpf embryos (Figure 6b(i)), some branching points were automatically added at the surface of the axonal shaft (Figure 6b(ii), arrow) and consequently wrongly increased the total length of the axon. We thus manually measured the length of the axonal shaft by selecting the axon with the measurement point module in the 3D mode of presentation (Figure 6b(iii)). However, no difference was observed between the two measurements of the axon length in 24 hpf and 48 hpf embryos (Figure 6c). We concluded that the increase in extra branching points that were mistakenly created due to the axon thickness was negligible.

We used the filament tracer module of the Imaris software, which permitted us to create a 3D reconstruction of motor neuron axons and, as such, to highlight the multiple axon branches observed in both 24 hpf and 48 hpf embryos. This representation enabled the visualization of branching points (Figure 6d, pink points). Quantification using Imaris software demonstrated that the branching length and the branching points, after normalization to axon length, were both significantly higher at 48 hpf than at 24 hpf (Figure 6e).

### 3.4. Length Measurement of Growing Motor Axons

**Figure 7:**
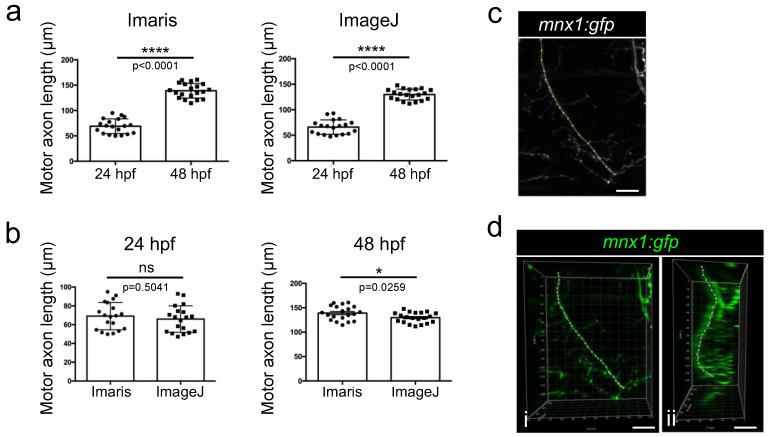
Comparison of Imaris and ImageJ methods for axon length quantification. (**a**) Quantification of motor axon length using measurement points on Imaris or ImageJ from 24 hpf (n = 19) and 48 hpf (n = 20) embryos. Statistical analysis was performed using Student *t*-tests. **** *p* < 0.0001. (**b**) Comparison of axon length measurements between the two methodologies in 24hpf or 48 hpf embryos. Statistical analysis was performed using Student *t*-tests. * *p* < 0.05 (**c**) Example of a manual selection of the nerve on a flattened (stack projection) 2D image of a motor nerve in a 48 hpf embryo obtained with imageJ in order to quantify it. Lateral view. (**d**) Example of measurement point selection on the same nerve performed with Imaris. Lateral view (i) and 90 degrees rotated view (ii). ns: non-significant. Error bars are SEM. Scale bar = 20 µm.

Each original stack of images was analyzed with both Imaris and ImageJ softwares. Point measurements of motor axon lengths were thus performed manually using the 3D views generated using Imaris software. Quantification showed a rapid expansion of the axonal shaft in zebrafish embryos that was accompanied by a significant doubling of length within 24 h of development (Figure 7a). This result was confirmed by manually measuring the length of the same axonal shafts from maximum intensity projection 2D images using the classical tool of segmented lines of the ImageJ software (Figure 7a). However, when comparing the length of the axons obtained with the two methods, a slight significant difference was observed between the two methods in 48 hpf embryos, axons being longer when using Imaris than when using ImageJ quantification (Figure 7b). Compared to the flattened 2D image obtained with ImageJ (Figure 7c), the Imaris software takes into account the full 3D shape of axons, as confirmed by a 90° rotation of the image that should improve measurements (Figure 7d).

## 4. Discussion

We described here an easy and fast new method to analyze and quantify axon morphology and branching. The analysis of intensity profiles led to the precise measurement of collateral numbers all along the length of the axonal shaft in the two dimensions (transversal or longitudinal). All the values could then be analyzed to determine the distribution of collaterals within a pre-selected area. Using this method, we quantitatively confirmed previous study-based observations suggesting that the ventral area of motor axons presents a higher number of collaterals than the dorsal area in 48 hpf embryos [7,13]. Moreover, quantification of the dorsal part of motor axons contains a higher number of collaterals in embryos 48 hpf compared to 24 hpf. This is in agreement with the joint development and maturation of axons, including the extension of secondary motor axons in the myotome at 34 hpf [9] and the maturation of their muscle targets [16].

While this new method using ImageJ required us to manually trace axonal shafts (about 30 s/axon), the overall analysis and quantification was then faster because it was possible to process by batch (only a few minutes were required to obtain the data for 20 images). For instance, Imaris software requires 5 min per image to manually select the axonal shaft and collaterals while unselecting objects out of the region of interest. The analysis with Imaris could also be a source of experimenter bias and, as such, needs to be performed blind.

Other plugins, such as NeuronJ (ImaScience) or Simple neurite tracer, may help the user trace the shape and length of axons and each individual collateral [17,18]. However, this process is time-consuming and needs the experimenter to select said collaterals. Thus, the final result will suffer from the bias of the experimenter, which we wanted to avoid as much as possible with our newly developed method.

It is noteworthy that the comparison between transverse and longitudinal profiles obtained from fluorescence intensity images or binary mask images yields slightly different results. Even though the former is easier to perform, the additional step of segmentation introduced by the production of mask images made the latter far more reliable. This has also been observed using an ImageJ plugin to measure neurite outgrowth and proliferation [19].

Still, our method presents certain limitations. The difference in the axon length values obtained with each method highlights the fact that our method is more suitable for the analysis and quantification of axons growing mostly within the image plane, in 2D. Moreover, our method focuses on the characterization of global branching defects; it does not categorize primary, secondary or higher order branches. Finally, the analysis with our script also requires sparse distribution of labeled neurons, as do all the automatic non-IA-based methods [15].

## 5. Conclusions

In conclusion, we have developed a new method to analyze axon arborization using ImageJ that is easy to perform and gives access to a large range of information, such as the distribution of collaterals along the axonal shaft. This method could be easily applied to other animal models, notably drosophila, as well as 2D and 3D cultured cells. It can also be used to study nerve regeneration, as the full recovery of the nerve can be assessed with our method by analyzing the distribution of axons along the nerve. Beyond that, it could be helpful to analyze and quantify branching morphogenesis, central to the development of various organs, including vasculature and lungs.

## Data Availability

The authors confirm that the data supporting the findings of this study are available within the article.

## Supplementary Materials

The following supporting information can be downloaded at https://www.mdpi.com/article/10.3390/mps6060116/s1. Figure S1: Median, min and max values obtained for mean fluorescence intensity/µm² calculated from the transverse and longitudinal profiles of 24 hpf and 48 hpf axons. Figure S2: Individual transverse and longitudinal profiles from 19 mask images of 24 hpf axons and 20 mask images of 48 hpf axons. Table S1: 24/48 hpf transverse profiles values. Table S2: 24/48 hpf longitudinal profiles values. Table S3: 24/48 hpf transverse profiles multiple *t*-test comparison title. Table S4: 24/48 hpf longitudinal profiles multiple *t*-test comparison. Table S5: 24/48 hpf transverse profiles values. Table S6: 24/48 hpf longitudinal profiles values. Table S7: 24/48 hpf transverse profiles multiple *t*-test comparison. Table S8: 24/48 hpf longitudinal profiles multiple *t*-test comparison.
