# Supplementary material for: Quantitative Image Analysis of Axonal Morphology in In Vivo Model
Source: Methods Protoc. 2023 Dec 1;6(6):116. doi: 10.3390/mps6060116 (PMC10745806; doi:10.3390/mps6060116)
Supplement: Supplementary file 1 [file mps-06-00116-s001.zip › Supplementary figures Nemoz-Billet et al.pdf]

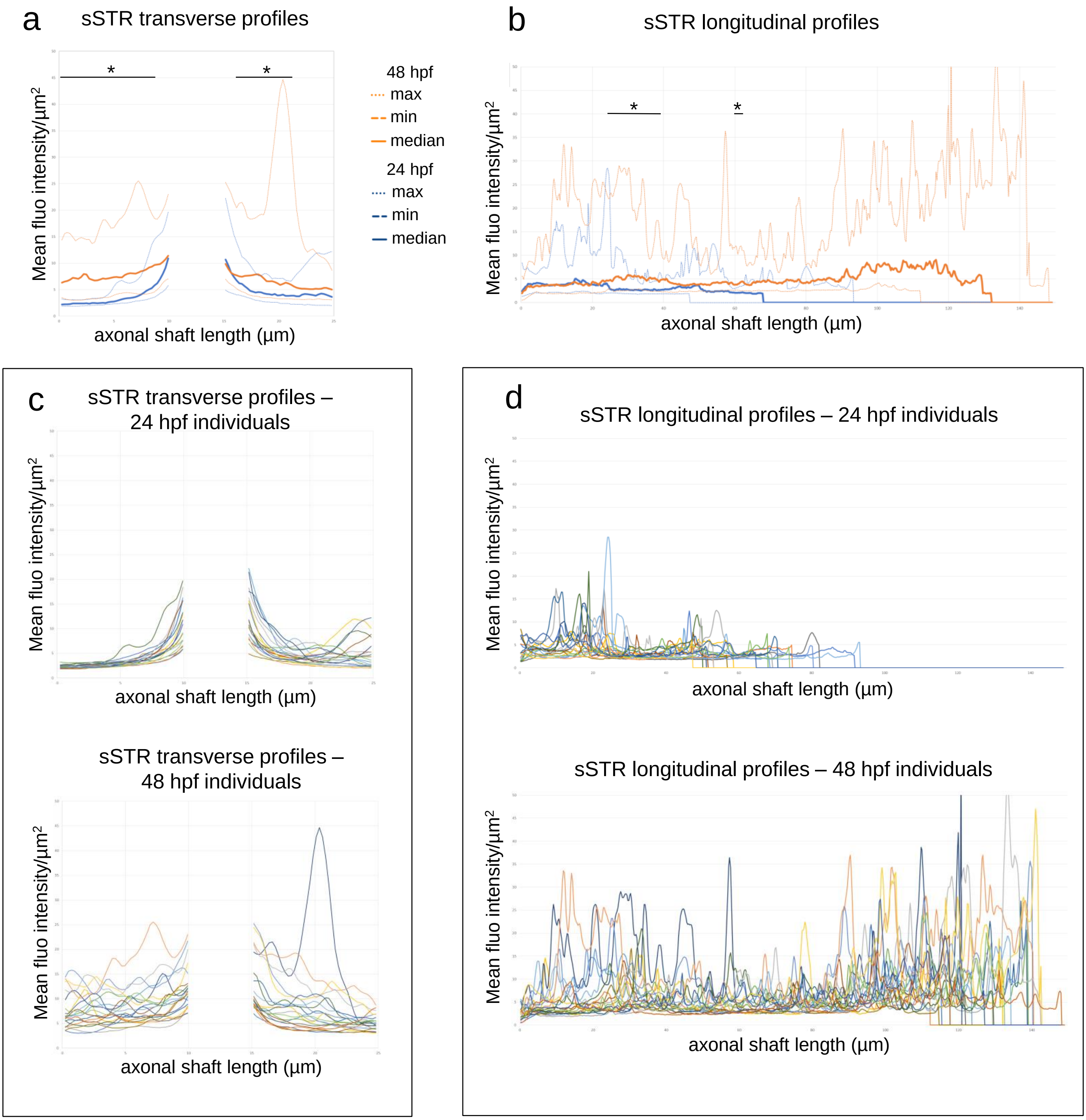

Figure S1. Median, min and max values obtained for mean fluorescence intensity / µm² (top panels) calculated from the transverse (a) and longitudinal (b) profiles of 24 hpf (dark blue) and 48 hpf axons (orange). *, significantly different profiles using Mann-Whitney tests corrected for multiple comparisons (individual pvalues in Tables S3 and S4).
Individual transverse (c) and longitudinal (d) profiles from 19 fluorescence intensity images of 24 hpf axons and 20 fluorescence intensity images of 48 hpf axons.

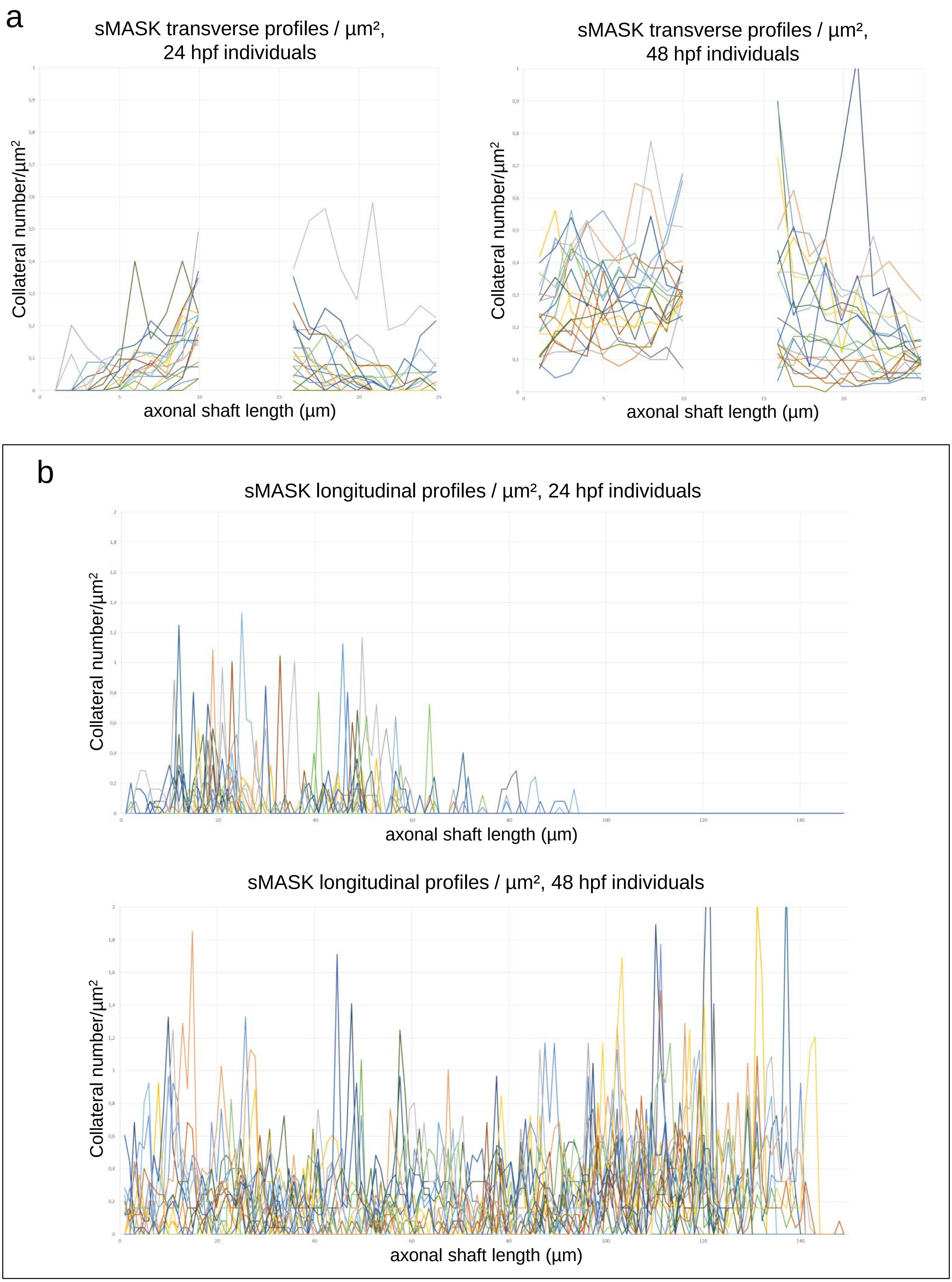

Figure S2. Individual transverse (a) and longitudinal (b) profiles from 19 mask images of 24 hpf axons and 20 mask images of 48 hpf axons.
